# The Distribution and Population Density of Bornean Tarsier, “*Tarsius Bancanus Borneanus* (Elliot)” in Secondary and Rehabilitated Forests of Universiti Putra Malaysia, Bintulu Sarawak Campus, Sarawak, Malaysia

**DOI:** 10.21315/tlsr2018.29.1.10

**Published:** 2018-03-02

**Authors:** Hani Nabilia Muhd Sahimi, John Keen Chubo, Marina Mohd. Top @ Mohd. Tah, Noor Bahiah Saripuddin, Siti Sarah Ab Rahim

**Affiliations:** 1 Department of Forestry Science, Universiti Putra Malaysia Bintulu Sarawak Campus, 97008 Bintulu, Sarawak, Malaysia; 2 Department of Crop Science, Universiti Putra Malaysia Bintulu Sarawak Campus, 97008 Bintulu, Sarawak, Malaysia; 3 Department of Biology, Faculty of Science, Universiti Putra Malaysia, 43400 Serdang, Selangor, Malaysia

**Keywords:** Behaviour, Distribution, Endemic, Morphology, Primate

## Abstract

*Tarsius bancanus borneanus* was first reported by Elliot in 1990 which an endemic species that can be found on the Island of Borneo consisting of Sabah and Sarawak of Malaysia, Brunei Darussalam and Kalimantan, Indonesia. This sub-species has been listed as a totally protected animal under the Sarawak Wild Life Protection Ordinance (1998) and vulnerable by the International Union for Conservation of Nature (IUCN). The present study was conducted at Universiti Putra Malaysia Bintulu Campus (UPMKB), Sarawak from October 2014 till March 2015. Through mark and recapture sampling covering an area of 37 ha of secondary forest patches and 7.13 ha of rehabilitated forest, a total of 16 tarsiers were captured using mist nets while one tarsier was recapture. The population density was 38 individuals/km^2^ was captured using mist nets in the secondary forest while 28 individuals/km^2^ was recorded for the rehabilitated forest. Using the catch per unit effort (net hour) method, the average time for capturing tarsiers in the secondary forest patches was 26.6 net hour per animal and 30.0 net hour per animal in the rehabilitated forest. The presented results provides information on the presence of tarsiers in both the secondary and rehabilitated forests of UPMKB, Sarawak, Malaysia which underlines the conservation value of these forested areas.

## Abstract (ru)

*Tarsius bancanus borneanus* (kera hantu) pertama kali dicam oleh Elliot pada tahun 1990 merupakan spesies endemik yang boleh ditemui di kepulauan Borneo yang terdiri dari Sabah dan Sarawak (Malaysia), Brunei Darussalam dan Kalimantan, Indonesia. Sub-spesies ini telah disenaraikan sebagai haiwan yang terlindung sepenuhnya di bawah Ordinan Perlindungan Hidupan Liar Sarawak (1998) dan disenaraikan sebagai spesies yang terdedah kepada kepupusan oleh IUCN. Kajian ini telah dijalankan di Universiti Putra Malaysia Kampus Bintulu Sarawak (UPMKB), dari Oktober 2014 hingga Mac 2015. Menerusi persampelan tangkap-lepas yang meliputi kawasan seluas 37 ha hutan sekunder dan 7.13 ha hutan yang dipulihara, sebanyak 16 ekor kera hantu telah ditangkap menggunakan jaring kabut manakala seekor kera hantu telah berjaya ditangkap semula. Kepadatan populasi kera hantu yang ditangkap menggunakan jaring kabut di dalam hutan sekunder adalah 38 individu/km^2^ manakala 28 individu/km^2^ dicatatkan bagi hutan yang dipulihara. Dengan menggunakan kaedah penangkapan per satu unit usaha (net-jam), masa purata bagi seekor kera hantu ditangkap di dalam hutan sekunder adalah 26.6 jam/haiwan dan 30 jam/haiwan di dalam hutan yang dipulihara. Keputusan membuktikan data mengenai kehadiran kera hantu di dalam kedua-dua hutan sekunder dan hutan yang dipulihara terutamanya di kampus UPMKB sekaligus menonjolkan nilai pemuliharaan kawasan hutan tersebut.

## INTRODUCTION

The name ‘tarsier’ is derived from the word ‘tarsal’ meaning ankle bone (Encyclopedia Britannica 2009). The unique characteristics of a tarsier include the capability of moving the head by almost 360 degrees, large eyes, long tarsal bone and very long hind legs which gives it the ability to search for prey and perform great leaps of up to 5–6 meters to catch its prey (MacKinnon & MacKinnon 1980). Tarsiers are restricted to the Southeast Asian island nations of Brunei, Indonesia, Malaysia, and the Philippines (Brandon-Jones *et al.* 2004). Tarsiers are found in a broad variety of habitats, including primary and secondary habitats, as well as certain habitats under human cultivation or use (MacKinnon & MacKinnon 1980; Crompton & Andau 1986). Conservation of primates has been inadequately acknowledged especially for tarsiers (Gursky *et al.* 2008). Studies on *Tarsius bancanus borneanus* have been very slow compared to the Philippines Tarsier (Brandon-Jones *et al.* 2004), as the species cannot be bred well in captivity and the specimen for the capture is low in number (Hellingman 2004). The whole species range of *Tarsius bancanus borneanus* occurrence as recorded by Brandon-Jones *et al.* (2004) was estimated to be more than 100,000 km^2^. Gursky *et al.* (2008) acknowledged that data on tarsier species and populations, especially its distribution is lacking. More regional studies should be conducted to obtain accurate data so that estimations on population density and other parameters can be scientifically recorded. According to the IUCN (2015), 30% of *Tarsius bancanus borneanus* habitat has been lost over the last 20 years. As scientific data for this sub-species is still lacking in Sarawak, it is high time to collect such data so that the existence of the species can be documented for future references. Thus, the aim of this study was to determine the species distribution, population density, average time of capture, including morphology and behavior sampling of *Tarsius bancanus borneanus* in the secondary and rehabilitated forests of Universiti Putra Malaysia Bintulu Campus (UPMKB), Sarawak, Malaysia.

## METHODS

### Study Sites

Universiti Putra Malaysia Bintulu, Sarawak Campus (UPMKB) is one of the few green lungs still left in the Bintulu Division (Norfahiah *et al.* 2012). Nowadays, the development in Bintulu is rapid and more forested area are being cleared for residential and industrial purposes. When the total area of primary and secondary forests decrease it may harm the population of wildlife in terms of habitat loss. Deforestation may cause wildlife to find other places for living and Nirwana Forest Reserve and the Mitsubishi Rehabilitation Forest of UPMKB is the only green place for them. *Tarsius bancanus borneanus* may receive a huge habitat loss impact and the population may decreased from day to day. There are several forest patches in UPMKB that can be classified as fragmented forest (secondary forest type). Fragmented forest resulted in the loss of habitat, formed isolated patches of forest and creating edge effects by breaking up forest areas (Bogaert 2000). According to Wade *et al.* (2003), forest fragmentation is a process that separates large areas of forest into smaller pieces. The campus area consists of three main forest patches: the Botanical Garden, Biopark, and a teaching forest known as the Reserve. The study site is located at the Nirwana Forest. Natural regeneration has occurred for years in the secondary forest of the Nirwana Forest Reserve. Species found on site are the dipterocarp (*Shorea* spp. and *Hopea* spp.) and non-dipterocarp *(Macaranga* spp., *Mallotus* spp.) species (Aiza *et al.* 2013). According to Aiza *et al.* (2013), the average diameter, at breast height, of the species ranged between 5.0 to 30.0 cm, while for the tree height, it was about 5.0 to 25.0 m. Rehabilitated forest can be defined as the renewal of forest crop through natural or artificial means (Yadav 2014). In this study, the regenerated forest area did not occur naturally but was man-made or planted. According to the Food and Agriculture Organization of the United Nations (FAO) 2010, planted forest is art of the forest regeneration which has two purposes: to protect and produce. Under the UPMKB context, the established planted forest acts as a protection instead of an area. Protective planted forest is an area that is established by way of a planting technique using native or introduced species (FAO 2010). There are four phases of rehabilitated forests in UPMKB, and only Phase I and II of the areas were used for this research. The rehabilitated forest is also known as the Malaysia Tropical Forest Regeneration Experimental Project which was a joint research project between Yokohama National University and Universiti Putra Malaysia, and sponsored by the Mitsubishi Corporation in 1990 (Aiza *et al.* 2013). In the rehabilitated forest several species were planted, such as *Shorea macrophylla, Shorea pinanga, Shorea palembanica, Shorea ovata, Shorea stereoptera, Dryobalanops aromatica* and *Hopea* spp. (Aiza *et al.* 2013). In the case of Bintulu town, the Nirwana Forest Reserve (a secondary forest) and the Mitsubishi Rehabilitation Forest (a rehabilitated forest) are the only two places available near UPMKB as alternative homes for wild life which are in close proximity to its natural habitat.

### Sampling

This study was conducted during the rainy season from October 2014 to March 2015. The sampling area covered 37 ha (0.37 km^2^) of secondary forest patches and 7.13 ha (0.0713 km^2^) of rehabilitated forest. In order to determine the location of tarsiers, several methods were applied such as reviewing secondary data from earlier study (Mahmad Yatim & Sylvia 2007; Nur Fadzlyn & Muhammad Shahrul Anuar 2009; Muhammad Hafiz & Siti Asmah 2010), sensing of urine marks on tree trunks and scouring for suitable habitats (Yustian 2006). During one pilot study conducted one of the field assistant found a urine mark on a tree left by a tarsier before disappearing to a higher level. The urine marks scent also was confirmed when one tarsier urinated at the time it was caught in the mist net.

### Data Collection

Tarsiers in this study were captured using mist nets via the mark and recapture method which have been used by Fogden (1974) and Crompton and Andau (1986). The size of the mist net used was 9–12 m long, 2.10–2.70 m wide with a mesh size of 16 mm^2^ (Yustian 2006). Nets were set up at 17:30 hours and closed after 23:30 h at the same location for at least three consecutive nights. To allocate the mist-nets, several plots have been established in the secondary and rehabilitated forests. Plots were set up randomly according to the area where the tarsiers have been captured according to previous study by Ahmad (2010). A total of 37 plots that were randomly selected near the forest edge of the secondary forest. For the rehabilitated forest, there were seven selected plots near the forest edge. The size for each plot is 20 × 50 m or 1 ha. Similar to the method used by Merker (2006), two to six mist-nets were set up inside the plot randomly after scanning the potential area to catch the tarsiers. In this research, more mist-nets were used to increase the chances of capturing the tarsiers. About 15 to 20 mist-nets were used per night in an area. Nets were inspected every 1 to 2 h to ensure that they were free from unwanted trapped birds, bats and insects (Yustian 2006). Tarsiers caught in the mist net were removed without using sedatives. Cotton gloves were used to avoid harming the tarsier. After being released from the net, tarsiers were kept in a cloth bag and parameters taken out to measure the morphology parameters. Three persons were required to assist in the measurement, recording and holding the tarsier. The tarsier was released after 10 min after all the measurement have been completed. Before being released at the place of capture, a temporary nail polish was applied to the hind leg nail for detection of recapturing. GPS reading for the capture location was recorded using GARMIN GPSmap 62s. All GPS coordinates were transferred to the Base Camp GARMIN Software for analysis. Data on elevation and distance from the main trail were also collected by using the same GPS map 62s for further analysis. There were two main trail (T1 and T2) located in this study i.e. T1 trail length was 810 m while T2 was 2694 m. Every distance where the tarsiers were captured and observed were marked to the nearest main trail. The distance of main trails depended on the accessibility to research areas. For behaviour observation, the ad libitum Sampling Method was used. Mann (2002) stated that it is possible to use more than one sampling methods for animal behaviour. In the ad libitum sampling method, the data was recorded according to the researcher encounter. Although not specific, it was useful for initial observation. It is also known as a typical field note (Mann 2002).

### Data Analysis

The population density for the mark and recapture method was estimated according to Brower *et al.* (1990), by dividing the number of observed/captured animals with the total area (*Di = ni/A*) while, the average of -catch per unit effort (net hours) was determined by dividing the capture effort hours with the number of captured animals using mist nets. The following morphological measurements were taken using a caliper and measuring tape: (1) tail length, (2) tuft length, (3) body length, (4) head and body length, (5) hindlimb length, (6) hindfoot length, (7) arm length, (8) forearm length, (9) hand length, (10) thumb diameter, (11) ear length. The morphological measurements and attachment procedure took approximately 20 minutes (Yustian 2007). Student t-tests were used to compare the means of the morphometric data. The testis volume was calculated using the formula: V = 4/3 π (0.5L)*(0.5W)^2^ (Fietz 1999). Morisita’s Index (Morisita 1959) was adopted to elicit the distribution pattern of tarsiers where Id = n * [(∑x^2^−∑x/(∑x)^2^−∑x)]; n is the number of sampled parcels and x_i_ the number of ramets or genets in each sampled parcel.

## RESULTS

### Population Density

Fourteen tarsiers were captured in the secondary forest using mist nets and only two tarsiers were captured in the rehabilitated forest. Only a single tarsier was recaptured in the rehabilitated forest during the entire research period. The population density per km^2^ estimation using the capture effort method for the secondary and rehabilitated forests was 38 and 28 individuals, respectively (Table 1).

### Average Time of Tarsiers Captured

Differences in the coverage of the research area influenced the average number of captured animal per capture effort (net hours). Only 14 individuals were captured within the 372 h spent to cover 37 ha of the secondary forest patches. Meanwhile, in the smaller area of the rehabilitated forest (7.13 ha) a total of 60 h was spent to capture two tarsiers. Thus, in the secondary forest, the average time to capture a tarsier was quantified as 26.6 net hours/animal while for the rehabilitated forest it was 30.0 net hours/animal (Table 1).

### Distribution

**Figure 1:**
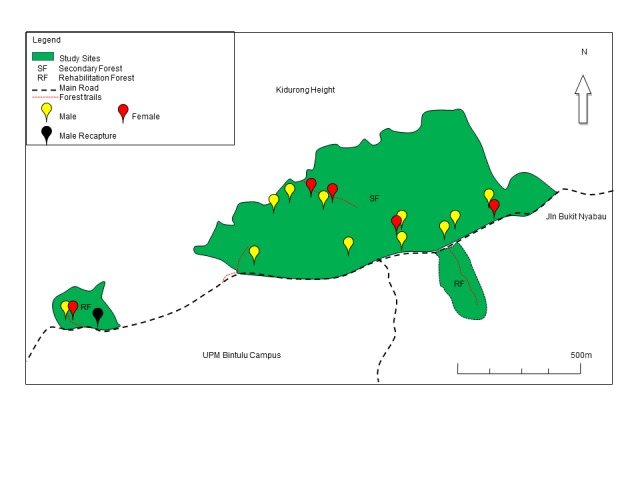
The distribution of tarsiers

The distribution of tarsiers can be seen in Fig. 1. Morisita’s Index has been widely used to determine the dispersion of individuals in a population (Morisita 1959). Morisita’s Index for the secondary forest was I_d_ = 4.88 (N = 14 tarsiers, n = 37 plots) and I_d_ = 7.00 (N = 2 tarsiers, n = 7 plots) for the rehabilitated forest. The Morisita’s Index recorded an I_d_ of more than 1.0, indicating that the distribution was patchy. Thus, in both sampling areas, the Morisita’s Index for dispersion indicated that the *Tarsius bancanus borneanus* was not randomly distributed.

### Morphological Measurement

The morphological measurement of *Tarsius bancanus borneanus* in the secondary and rehabilitated forests are shown in Table 2. An independent *t*-test was conducted to compare the morphological data between male (N = 10) and female tarsiers (N = 4) captured in the secondary forest. The measurement of the length of the hind limbs (thighs) of male tarsiers were found to be significantly longer (Mm = 71.10 mm, SDm = 4.31) than the female tarsiers (Mf = 62.30 mm, SDf = 8.85) with t = 2.586 and p = 0.024. Range survey, morphological measurements and independent *t*-tests were not conducted for the rehabilitated forest as only one male and one female tarsier were captured. *Tarsius pumilus* and *Tarsius bancanus borneanus* have the same preference to live nearby the main trail. According to Merker and Muhlenberg (2000), *Tarsius dianae* in Sulawesi, Indonesia also have been found near to the human area with the range of distance between 80–133 m. Most of the studies suggested that tarsiers can easily be observed or captured nearby the forest edges. This situation occurs because of the food resources availability such as insect which were more abundant nearby the forest edges (Grow *et al.* 2013).

### Morphology

Payne *et al.* (1985) reported that the weight range for this subspecies were between 86.00 g–135.00 g. Mahmad Yatim and Sylvia (2007) recorded the weight of tarsiers ranging between 141.1 g–150.2 g which was more than the maximum weight recorded by Payne *et al.* (1985).

### Testis Volume

A total of 11 male tarsiers were caught and measurements of testis length and width were recorded (Table 3). The testis volume of male tarsiers varies from 161.0 mm^3^–670.3 mm^3^. The r^2^ value was 0.828 indicating a high degree of correlation occurred between the testis volume and body weight of male tarsiers with about 81.1% of total variation for testis volume can be explained by the body weight. The relationship between the testis volume and body weight of male tarsiers in both the secondary and rehabilitated forests was highly significant (*p* < 0.01).

### Behaviour

Four tarsiers were spotted through the observation process. A tarsier was detected on a tree trunk at an observation height of more than two metres (25% of total observation) while three spotted tarsiers were observed below two meters (75% of total observation). The observation data also revealed the movement of the tarsiers. Observations conducted on four tarsiers recorded that the subspecies preferred small diameter trees to leap from i.e. mean 3.1 cm in the present study. Based on observation, tarsiers will take a few minutes to leap and usually they turn their heads left and right to seek for a suitable tree to escape. Such behaviour was performed by the tarsiers in the secondary forest. Tarsiers do not perform rapid disturbance behaviour when light is shown on them.

Five urine marks were detected in several lines within the research areas. The first urine mark was found at 16:00 h during the setting up of the mist nets. The other three urine marks were detected between 19:30 to 20:30 h while one urine mark was sensed at 22:00 h. The smell of urine marks can be easily detected near the mist nets area where the calls were heard. Data on vocalisation signals was insufficient for comparison. The calls can be described in three short phrases, ‘tsit-tsit-tsit’ similar as described by Niemitz (1979).

## DISCUSSION

### Population Density

The population density of *Tarsius bancanus borneanus* in the secondary forest in this study was higher (38 individuals/km^2^) compared to the rehabilitated forest but lower compared to other literature reports. Earlier sampling conducted in 2006 until 2009 recorded nine individuals of *Tarsius bancanus borneanus* in the area (Norfahiah *et al.* 2012) with one individual located in the Mitsubishi Rehabilitated Forest and another eight individuals in the Nirwana Forest Reserve. The number of *Tarsius bancanus borneanus* in the rehabilitated forest was only 28 individuals/km^2^. Niemitz (1979) noted that the population density of *Tarsius bancanus borneanus* in Sarawak was less than 80 individuals/km^2^. Crompton and Andau (1987) reported that the population density of the same subspecies at the secondary forest of Sepilok Forest Reserve in Sabah was merely 14–20 individuals/km^2^. Meanwhile, Blackham (2005) stated that the population density of *Tarsius bancanus borneanus* in the peat swamp forest of Central Kalimantan was 0.003 individuals/km^2^ using distance sampling. Another comparison which can be made was with the *Tarsius bancanus saltator* in small-scale plantations and logging areas of Belitung Island where Yustian (2006) reported a population density of only 25–26 individuals/km^2^. It was stipulated the population density of tarsiers could have been affected by the location and topography of study sites. Blackham (2005) noted that habitat types could also influence population density estimations. In this study, sampling during the wet season and dry season was not conducted. Seasonality is one of the factors affecting the presence of tarsiers. From previous study, it was shown that seasonality gave impact on insect abundance and behavior of *Tarsius spectrum* in Tangkoko-Dua Saudara Nature Reserve, North Sulawesi, Indonesia (Gursky 2000). However, according to Merker (2006), seasonality did not influence the ranging patterns of *Tarsius dianae* in Lore-Lindu National Park, Central Sulawesi, Indonesia. There were differences between the result for seasonality between Gursky (2000) and Merker (2006). According to Gursky (2000), the food resources during the dry season was lacking thus forcing tarsiers to use most of their time on foraging than other activities.

### Average Time of Tarsiers Captured

This research recorded 26.57 net hours to trap tarsiers in the secondary forest and 30.00 net hours in the rehabilitated forest. A study by Ahmad (2010) did not state the average time for tarsiers to be captured. Distance sampling was used by Blackham (2005) but was not comparable to this research due to the use of different sampling methods. Yustian (2006) who used the same method in his research on *Tarsius bancanus saltator* stated that 109.18 net hours was used to catch a tarsier in a secondary forest comprising of a small pepper farm and logging activites. A total of 51.06 net hours was recorded to catch a tarsier in a secondary forest with logging activities and surrounded by oil palm plantation (Yustian 2006).

### Distribution

Grow *et al.* (2013) reported that the spatial distribution of pygmy tarsiers in the highland mossy cloud forest of Mt. Rore Katimbu (2000–2300 m a.s.l), Lore Lindu National Park, central Sulawesi Indonesia showed some patchy distribution patterns (Id = 2.55) similar to the present study. The availability of pygmy tarsier (*Tarsius pumilus*) in the highlands and *Tarsius bancanus borneanus* in the lowlands, indicated that altitude does impact the distribution of tarsiers which are usually found near forest edges (Grow *et al.* 2013). Gursky (2000) noted that forest edges attract tarsiers because of food sources such as airborne insects which can be easily be found.

The altitude factor did affect the distribution and both tarsiers found nearby the forest edges (Grow *et al.* 2013). Primates usually adapt to the effects of altitude through foraging efficiency and competition by adjusting to the distribution and size of the population (Grow *et al.* 2013). According to Grow *et al.* (2013), primates that occupy the higher altitude face different type of ecological environment than those occupying the lower altitude. Tarsiers were captured and observed within a distance of 10.0 m to 171.0 m from the main road. These distances were different from those recorded by Ahmad (2010) who noted a distance of between 36.52 m to 88.42 m from the main road. The distance range in the present study seemed to be further from previously recorded data for the same area. Such finding might be due to the land clearing activities occurring on the opposite site of the forest. Observation on the forest edge near the road side also noted more food resources such as insect (according to the sound and observation). Changes in the distance from the main trail year by year can be related to the change in food location (Ahmad 2010).

A study by Grow *et al.* (2013) found that *Tarsius pumilus* sleeping sites in Mount Rore Katimbu, Lore Lindu National Park, Central Sulawesi, Indonesia were ranging between 30–164 m from the main road indicating that *Tarsius pumilus* may have the same preference to live nearby the main trail as *Tarsius bancanus borneanus*. Similarly, Merker and Muhlenberg (2000) indicated that *Tarsius dianae* in Sulawesi, Indonesia with a distance between 80 m–133 m. Most of the studies suggested that tarsiers can be easily observed or captured nearby the forest edges. This situation occurs because of the food resources availability such as insect which are more abundant nearby the forest edges (Grow *et al.* 2013).

### Morphology

Payne *et al.* (1985) reported that the weight range for this subspecies were between 86.00–135.00 g. Mahmad Yatim and Sylvia (2007) recorded the weight of tarsiers ranging between 141.1 g–150.2 g which was more than the maximum weight recorded by Payne *et al.* (1985). An earlier survey by Ahmad (2010) in UPMKB recorded four tarsiers with heavier weights between 80.00 g–154.80 g. In comparison, the present study captured no tarsier below the minimum weight range as recorded by Payne *et al.* (1985), although nine tarsiers weighed more than 135.00 g. Higher weight recorded in this study could be related to the breeding season (Fogden 1974). According to Yustian (2007), the differences in body weight might due to the different reproductive status (pregnant, lactative or non-reproductive). With reference to the sex ratio, more male tarsiers were captured than females in this study. A research conducted in Sabah, Malaysia reported that the number of male tarsiers captured was higher as the home range size was larger than female tarsiers (Crompton & Andau 1987). Yustian (2009) further confirmed in his research of *Tarsius bancanus saltator* on Belitung Island, that the home range of male tarsiers was larger than the females because of differences in habitats.

The female tarsiers used the home range only to search for food while the male tarsiers used them for territorial and mating purposes. Both captured male and female tarsiers showed a positive relationship in terms of body weight and hindlimbs (thigh). The captured female tarsiers showed a steeper line slope indicating a stronger relationship between body weight and hindlimbs (thigh). The hindlimbs of tarsiers are shaped for its locomotor behaviour such as clinging and leaping (Anemone & Nachman 2003). This relates to the home range activities of male and female tarsiers. The locomotion of male tarsiers is wider than the females according to their home range (Yustian *et al*. 2009). Thus, when the tarsier is moving actively, it will affect the shape and size of its hindlimbs. This is the reason why male hindlimbs are longer than female hindlimbs. According to Grow *et al.* (2013) there is a positive relationship between body mass and hindlimbs of lowland tarsiers which is similar in the present study.

### Testis Volume

This study was conducted during the breeding season and the results of testis volume was quite different from a study by Wright *et al.* (2003) where the body weight of *Tarsius bancanus borneanus* can explained 0.15% of the variance in average testis volume (*p* < 0.91) during the non-breeding season while during the breeding season the percentage increased to 2.57% (*p* = 0.66). This differed from the *Tarsius syrichta* of the Visayan Islands, Philippines. During the non-breeding season, 36.5% body weight of *Tarsius syrichta* can be explained by the average testis volume (*p* < 0.005). However, during the breeding season, only 30.3% of body weight can be explained by the variance in average testis volume with *p* = 0.08 (Wright *et al.* 2003).

The differences in testis volume may depend on the length of the breeding season i.e. three months (September–November) for *Tarsius syrichta* and six months (August–January) for *Tarsius bancanus* (Wright *et al.* 2003). The sample size for each study could also contributed to the differences i.e. only two male *Tarsius bancanus* were caught by Wright *et al.* (2003) as compared to 11 males caught in this study.

### Behaviour

The mean for the observation height was 1.8 m. From four observations, the recorded tree diameters were 2.8 cm, 2.9 cm, 3.1 cm and 3.5 cm with a mean of 3.1 cm. The percentage of total observation for diameter size below 3.0 cm was 50% and for the diameter of more than 3.0 cm was also 50%. Crompton and Andau (1986) recorded an observation height of 0.89 meter with 76.3% below 1.0 meter. Similarly, Crompton and Andau (1986) reported that the mean diameter size used by tarsiers for leaping was 2.8 cm at the Sepilok Forest Reserve, Sabah. They also indicated that when small diameter trees were lacking in the area, tarsiers could still leap onto bigger diameter trees but their movements would be shaky and sometimes they slipped. Once the tarsiers stabilised their bodies, they will find a way to cling onto smaller trees nearby. Multiple flash photographs of leaping technique taken by Crompton and Andau (1986) in Sepilok, Sabah were also observed in the present study. The time gap for the leaping behaviour from one tree to another is fast, far enough and cannot be easily detected at night.

### Influence of Habitat Type on Population Density

Blackham (2005) suggested that habitat can influence the population density estimation. Crompton and Andau (1986) reported that tarsier prefer the secondary forest rather than the undisturbed or primary forests. Similar findings were reported for the Dian’s tarsier in Kamarora, Sulawesi, Indonesia where higher population density was found in the secondary forest rather than the primary forest (Gursky 2015). According to Gursky (2015), tarsiers population density is known to change substantially with both altitude and habitat type. In this study, there were differences in habitat types of the secondary forest which was regenerated naturally after logging while the rehabilitated forest was planted artificially. The topographical characteristics of the secondary and rehabilitated forests were different. In terms of slopes and elevation, the secondary forest has a complex terrain compared to the rehabilitated forest which consists of huge flat area. This factor can be related to the niche of the tarsier itself. The niche was influenced by the randomly distributed vegetation of the secondary forest and the systematic plantation in the rehabilitated forest thus influencing the locomotion especially leaping technique of the tarsier (Gebo 2014). Tarsiers are known to have the ability to move in dense vegetated area (King 2015). In this study, secondary forest was denser than the rehabilitated forest.

## CONCLUSION

This research confirms the presence of *Tarsius bancanus borneanus* in the secondary and rehabilitated forests of UPMKB, Sarawak, Malaysia. In order to observe *Tarsius bancanus borneanus* as a totally protected animal in Sarawak, efforts to conserve its natural habitat is necessary. As areas surrounding the campus are being developed, conservation measures and strategies must be put in place to prevent the extinction of the species. The secondary and rehabilitated forests are part of the of education forest for UPMKB student. Therefore, learning about this protected species should be highlighted so that more information can be channeled in order to prevent this animal from extinction. The forest boundaries within this area should be maintained to prevent any encroachment of the wildlife habitat.

## Figures and Tables

**Table 1:** Population density of tarsiers in the secondary and rehabilitated forests and average captured animal per capture efforts (nets-hour).

Habitat type	Total land (km^2^)	Total research area (km^2^)	Captured animal	Population density per km^2^	Capture effort (nets-hours)	Average (nets-hours/animal)
Secondary forest	1.60	0.37	14	38	372	26.57
Rehabilitated forest	0.37	0.07	2	28	60	30.00

**Table 2:** Morphometries of *Tarsius bancanus bomeanus* in secondary forest and rehabilitated forests

ID	W(g)	TLL	TFL	BL	HBL	HLL (lower leg)	HLL (thigh)	HFL	AL	FL	HL	EL (L)	EL (W)	TD (Hand)	TD (foot)
Secondary forest															

Male (N = 10)															
Mean/SD	130 ± 19.44	221.9 ± 15.79	72.9 ± 8.44	103.1 ± 10.31	148 ± 12.18	75.2 ± 5.69	71.1 ± 4.31	70.5 ± 4.48	34.9 ± 3.98	45.0 ± 3.02	48.4 ± 4.17	23.7 ± 3.30	20.8 ± 2.70	12.3 ± 3.13	21.5 ± 6.06
Min	100	204	60	87	126	64	64	62	31	41	41	18	18	8	16
Max	160	260	89	120	169	86	77	78	44	49	54	31	27	18	34
Female (N = 4)															
Mean/SD	135 ± 25.17	231 ± 11.69	70.5 ± 7.77	100.3 ± 5.50	142.5 ± 5.45	77.0 ± 6.88	62.3 ± 8.85	69.8 ± 7.14	32.8 ± 4.99	43.8 ± 4.27	48.5 ± 5.26	25.8 ± 3.10	22.5 ± 2.08	14.8 ± 4.92	25.8 ± 6.29
Min	100	219	59	95	135	67	55	61	26	39	44	23	20	11	21
Max	160	242	76	105	147	82	73	78	38	49	56	30	25	22	35

Rehabilitated Forest															

Male	140	205	55	130	154	80	65	67	25	48	50	30	20	12	27
Female	160	210	60	146	173	77	53	85	55	74	54	20	11	14	24

* Note: W = weight; TLL= tail length; TFL = tuft length; BL = body length; HBL= head and body length; HLL= hindlimb length; HFL=hindfoot length; AL = arm length; FL = forearm length; HL= hand length; EL (L) = ear length; EL (W) = ear width; TD= thumb diameter.

** Note: All measurements were in (mm) except weight (g)

**Table 3:** Testis volume and body weight of *Tarsius bancanus borneanus* in secondary and rehabilitated forests.

No. of individuals	Month of capture	Average testis volume (TV) (mm^3^)	Body weight (BW) (g)	TV/BW
SFM1	October 2014	207.6	130	1.6
SFM2	December 2014	670.3	140	4.8
SFM4	December 2014	333.8	150	2.2
SFM5	December 2014	593.8	120	4.9
SFM6	January 2015	255.5	120	2.1
SFM7	January 2015	469.2	110	4.3
SFM8	January 2015	273.4	150	1.8
SFM9	January 2015	414.2	120	3.5
SFM10	February 2015	157.1	100	1.6
SFM11	February 2015	502.7	160	3.1
RFM3	December 2014	161.0	140	1.2
SD		175.7	18.7	

* Note: SF = Secondary Forest; RF = Rehabilitated Forest

## References

[b1-tlsr-29-1-139] Ahmad ZA (2010). Population density of Western Tarsier, *Tarsius bancanus* in Nirwana forest, Universiti Putra Malaysia Bintulu Sarawak Campus. Unpublished undergraduate dissertation.

[b2-tlsr-29-1-139] Aiza SJ, Arifin A, Hazandy A, Mohd Hadi A, Trevor SB, Shamshuddin J, Nik Muhamad M (2013). Assessing soil fertility status of rehabilitated degraded tropical rainforest. American Journal of Environmental Science.

[b3-tlsr-29-1-139] Anemone RL, Nachman BA, Wright PC, Simons EL, Gursky S (2003). Morphometrics, functional anatomy, and the biomechanics of locomotion among Tarsiers. Tarsiers: Past, present and future.

[b4-tlsr-29-1-139] Blackham G (2005). Pilot survey of nocturnal primates, *Tarsius bancanus borneanus* (Western tarsier) and *Nycticebus coucang menagensis* (Slow loris) in peat swamp forest, Central Kalimantan, Indonesia. Unpublished MSc dissertation.

[b5-tlsr-29-1-139] Bogaert J (2000). Quantifying habitat fragmentation as a spatial process in a patch-corridor matrix landscape model. Unpublished PhD dissertation.

[b6-tlsr-29-1-139] Brandon-Jones D, Eudey AA, Geissmann T, Groves CP, Melnick DJ, Morales JC, Shekelle M, Stewart CB (2004). Asian primate classification. International Journal of Primatology.

[b7-tlsr-29-1-139] Brower J, Zar J, Ende C (1990). Field and laboratory methods for general ecology.

[b8-tlsr-29-1-139] Crompton RH, Andau PM (1986). Locomotion and habitat utilization in free-ranging *Tarsius bancanus*: A preliminary report. Primates.

[b9-tlsr-29-1-139] Crompton RH, Andau PM (1987). Ranging, activity rhythms, and sociality in free-ranging *Tarsius bancanus*. International Journal of Primatology.

[b10-tlsr-29-1-139] Encyclopedia Britannica (2009). Tarsier.

[b11-tlsr-29-1-139] Food and Agriculture Organization (FAO) (2010). Planted forest.

[b12-tlsr-29-1-139] Fietz J (1999). Mating system of *Microcebus murinus*. American Journal of Primatology.

[b13-tlsr-29-1-139] Fogden MPL (1974). A preliminary field study of the western tarsier, Tarsius bancanus horsefield in the prosimian biology.

[b14-tlsr-29-1-139] Gebo DL (2014). Primate comparative anatomy.

[b15-tlsr-29-1-139] Grow N, Gursky S, Duma Y (2013). Altitude and forest edges influence the density and distribution of Pygmy Tarsiers (*Tarsius pumilus*). American Journal of Primatology.

[b16-tlsr-29-1-139] Gursky S (2000). The effects of seasonality on the behavior of an insectivorous primate *Tarsius spectrum*. International Journal Primatol.

[b17-tlsr-29-1-139] Gursky S, Shekelle M, Nietsch A, Shekelle M, Maryanto I, Groves C, Schluze H, Fitch-Snyder H (2008). The conservation status of Indonesia’s Tarsiers. Primates of the oriental night.

[b18-tlsr-29-1-139] Gursky S (2015). The spectral Tarsier: Primate field studies.

[b19-tlsr-29-1-139] Hellingman J (2004). The Philippine tarsier.

[b20-tlsr-29-1-139] IUCN (2015). The IUCN red list of threatened species.

[b21-tlsr-29-1-139] King JE (2015). Primate behavior and human origin.

[b22-tlsr-29-1-139] MacKinnon J, MacKinnon K (1980). The behavior of wild spectral tarsiers. International Journal of Primatology.

[b23-tlsr-29-1-139] Mahmad Yatim A, Sylvia L (2007). Tinjauan populasi mamalia kecil plot 16, Hutan Simpan Nirwana, UPMKB. Unpublished thesis.

[b24-tlsr-29-1-139] Mann J (2002). Cetaceans societies: Field study of dolphins and whales.

[b25-tlsr-29-1-139] Merker S (2006). Habitat-specific ranging patterns of Dian’s tarsiers (*Tarsius dianae*) as revealed by radiotracking. American Journal of Primatology.

[b26-tlsr-29-1-139] Merker S, Muehlenberg M (2000). Traditional land use and tarsiers: Human influence on population densities of *Tarsius dianae*. Folia Primatologica.

[b27-tlsr-29-1-139] Morisita M (1959). Measuring of the dispersion and analysis of distribution patterns. Series E Biology.

[b28-tlsr-29-1-139] Muhamad Hafiz AG, Siti Asmah M (2010). Kajian kepelbagaian dan kelimpahan spesies mamalia kecil di hutan ladang dan hutan nirwana. Unpublished thesis.

[b29-tlsr-29-1-139] Niemitz C (1979). Results of a field study on the western tarsier (*Tarsius bancanus borneanus*) Horsfield captivity: Evidence for a slow life history and nonmonogamous mating system. International Journal of Primatology.

[b30-tlsr-29-1-139] Norfahiah M, Azema I, Marina MT, Zakaria M (2012). Status and distribution of non-volant small mammals in Universiti Putra Malaysia Bintulu Sarawak Campus (UPMKB). Pertanika Journal of Tropical Agriculture Science.

[b31-tlsr-29-1-139] Nur Fadzlyn R, Muhammad Shahrul Anuar H (2009). Kajian kepelbagaian mamalia kecil di hutan nirwana, UPMKB. Unpublished thesis.

[b32-tlsr-29-1-139] Ong KH, John KC, Roland KJH, Marina MT (2008). Protecting of the last frontier: The role of Universiti Putra Malaysia Bintulu Campus in Biodiversity Conservation.

[b33-tlsr-29-1-139] Payne J, Francis CM, Phillips K (1985). A field guide to the mammals of Borneo.

[b34-tlsr-29-1-139] Rowe N (1996). The pictorial guide to the living primates.

[b35-tlsr-29-1-139] Wade TG, Riitters KH, Wickham JD, Jones KB (2003). Distribution and causes of global forest fragmentation. Conservation Ecology.

[b36-tlsr-29-1-139] Wright PC, Pochron ST, Haring DH, Simons EL, Wright PC, Simons EL, Gursky S (2003). Can we predict seasonal behavior and social organization from sexual dimorphism and testes measurements?. Tarsiers: Past, Present and Future.

[b37-tlsr-29-1-139] Yadav BKV (2014). Natural regeneration.

[b38-tlsr-29-1-139] Yustian I (2006). Population density and the conservation status of Belitung’s Tarsier. *Tarsius bancanus saltator* on Belitung Island. Indonesia. South Sumatera, Indonesia (Unpublished thesis).

[b39-tlsr-29-1-139] Yustian I (2007). Ecology and conservation status of Belitung’s Tarsier Tarsius bancanus on Belitung Island, Indonesia.

[b40-tlsr-29-1-139] Yustian I, Merker S, Muehlenberg M (2009). Luas daerah jelajah dan estimasi kepadatan populasi *Tarsius bancanus saltator* di Pulau Belitung. Jurnal Biologi Indonesia.

